# Delayed re-bleeding after removal of a radial arterial cannula

**DOI:** 10.1186/s40981-018-0202-1

**Published:** 2018-09-11

**Authors:** Natsumi Hatanaka, Kumiko Tanabe, Mayuko Yamada, Hiroki Iida

**Affiliations:** Department of Anesthesiology and Pain Medicine, Gifu University Graduate School of Medicine, Gifu, 501-1193 Japan

**Keywords:** Arterial cannulation, Complication, Delayed re-bleeding, Hematoma

## To the editor

Hematoma after arterial cannulation is not so very rare but tends to occur during cannulation and immediately after decannulation. We report a case of delayed re-bleeding after the removal of a radial arterial cannula.

### Case presentation

**Fig. 1:**
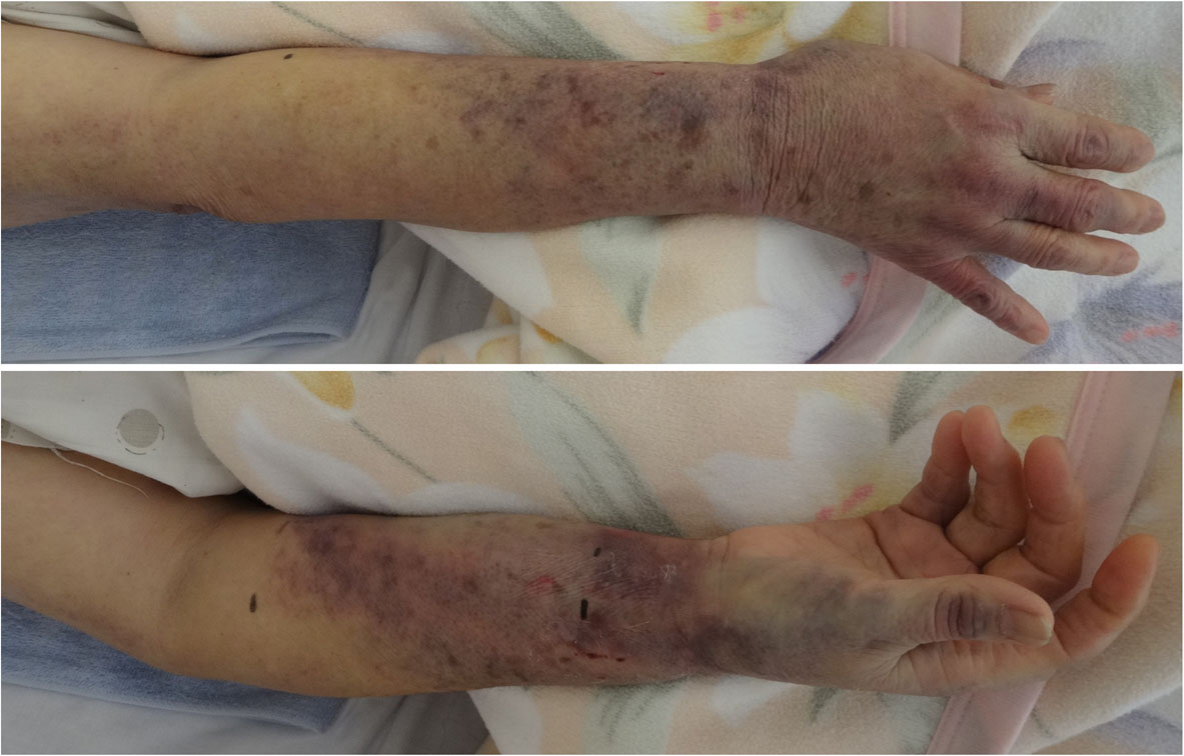
A picture of the right forearm. Two days after the operation, internal hemorrhaging and edema from the fingers to the middle of the upper arm were noted. The right forearm was stiff and swollen

A 74-year-old woman underwent revision total arthroplasty under general anesthesia with epidural anesthesia. She had no comorbidities and regularly took loxoprofen sodium hydrate. Examinations before the operation had no abnormal findings. Her right radial artery was punctured and cannulated uneventfully with a 22-gauge BD Insyte™ IV Catheter (Japan Becton, Dickinson and Company). The amount of bleeding was 320 mL, and she received 160 mL of autologous fresh-frozen plasma. The arterial cannula was immediately removed at the end of anesthesia. An anesthesiologist achieved hemostasis at the punctured site with 5 min of compression by hand. She exited the operation room with a hemostasis device (STEPTY; Nichiban Co., Ltd., Tokyo, Japan) over the puncture site. The device was removed 2 h after the operation, when her bleeding was confirmed to have stopped. She was observed every 2 h without re-bleeding at the puncture site, and her systolic blood pressure was kept under 120 mmHg. A nurse noted bleeding from the puncture site 13 h after the operation. She compressed the site by hand and called a duty doctor, who advised watchful waiting. They confirmed the stop of bleeding and noticed slightly edema and internal hemorrhaging around the puncture site. The next day, internal hemorrhaging and edema from the fingers to the middle of the upper arm were noted (Fig. 1). Her right forearm was stiff and swollen, but her hand movement was not restricted. She noted no paralysis or paresthesia. The pulse oximeter detected normal waves on her right fingers. Based on these symptoms, compartment syndrome was not suspected. The circumferences of her forearm at two points (proximal wrist joint and distal elbow) were checked eight times over 2 days, with no increase in size noted. The internal hemorrhaging and edema were gradually diminished.

### Discussion

Indwelling arterial catheters is generally a safe procedure with few serious complications [1, 2]. The radial artery is the most common site for cannulation because of a low rate of associated complications [3], although hematoma is occasionally noted. The incidence rate of transradial puncture-related hematoma is 14.4% [1], which is higher than that of hematoma related to cardiac catheterization for angiography or percutaneous coronary intervention (PCI) (0.5% to 13%) [4], even though the cannulas used for angiography and PCI are larger than those used in the operating room and patients frequently receive antiplatelet agents after PCI [3]. Patients who are older and have a lower body mass index or body weight and thinner skin folds are reportedly at risk of developing hematoma after transradial catheterization [4].

The radial arterial puncture site is usually manually compressed at the time of decannulation. However, basic questions such as how long and strongly compression should be performed remain unclear.

### Conclusion

We should check for hemostasis frequently in cooperation with the ward staff.

## Abbreviation

PCI: Percutaneous coronary intervention
